# Sociodemographic Determinants of Physical Activity and Sport Participation among Women in the United States

**DOI:** 10.3390/sports8070096

**Published:** 2020-07-02

**Authors:** Jennifer R. Pharr, Nancy L. Lough, Angela M. Terencio

**Affiliations:** 1 School of Public Health, University of Nevada Las Vegas, Las Vegas, NV 89154, USA; 2 College of Education, University of Nevada Las Vegas, Las Vegas, NV 89154, USA; nancy.lough@unlv.edu; 3 School of Integrated Health Sciences, University of Nevada Las Vegas, Las Vegas, NV 89154, USA; terencio@unlv.nevada.edu

**Keywords:** sociodemographic characteristics, physical activity, sports, women, United States

## Abstract

Regular physical activity and sport participation have been shown to improve women’s health; however, research has found that better health is associated with sport participation. Little is known about the sociodemographic determinants of physical activity among women, especially among the different subcategories of physical activity (sport, conditioning exercise, recreation, and household tasks). Because of the added health benefits associated with sport participation, the purpose of this study was to examine the sociodemographic determinants among subcategories of physically active women in the United States by analyzing Behavioral Risk Factor Surveillance System (BRFSS) data. We used data from the 2017 national BRFSS survey to conduct this secondary data analysis. Participants were asked an extensive set of questions about their physical activity. Seventy-six different activities were identified and categorized as either sport, recreation, conditioning exercise, or household tasks. Weighted descriptive statistics were performed to describe the sociodemographic determinants of the four physical activity subcategories, including age, income, education, employment, and race/ethnicity. There were significant differences in all sociodemographic variables among the four subcategories of physical activity. Women who participated in sport were more likely to be in the younger age groups; however, physical activity declined among all subcategories beyond the age of 64. Women who participated in sports were more diverse, likely to be employed, and college graduates compared to the other subcategories. Women who participated in recreational or household tasks were more likely to meet the criteria to be categorized as highly active; however, they exercised at a lower intensity. The sociodemographic characteristics of physical activity and sport participation can be used to create promotional strategies to increase physical activity and improve fitness and health among women who tend towards participation, and also to change programs to accommodate women from other sociodemographic groups.

## 1. Introduction

Regular physical activity and sports participation are associated with improved health and wellbeing [1,2,3,4,5,6,7]. When compared to adults who are physically inactive, those who achieve recommended levels of activity have a reduced risk of cardiovascular disease, including stroke or heart attack, diabetes, obesity, high cholesterol, high blood pressure, depression and cognitive impairment, and some forms of cancer [2,5,6,7,8]. They are also more likely to have increased bone density, pulmonary function, a sense of wellbeing, and better mood [2,3,5,9] Additionally, a lack of physical activity can lead to higher odds of cardiovascular mortality and all-cause mortality [10]. Specific to women, achieving recommended amounts of physical activity is associated with a reduced risk of metabolic syndrome, cardiovascular disease, and osteoporosis and an improved perception of health status and feelings of vitality [5,11,12,13,14,15,16]. For example, there are significantly fewer non-fatal or fatal heart attacks among women who walk briskly for three or more hours per week compared to women who are inactive [16].

The Department of Health and Human Services (DHHS) recommends that adults get 150 or more minutes a week of moderate-intensity aerobic exercise, or 75+ min a week of vigorous-intensity aerobic exercise, or a combination of the two (moderate and vigorous) [5]. Despite the numerous health benefits associated with physical activity, in the U.S., only 53.7 percent of adults over the age of 18 participate in the recommended amount of aerobic physical activity, and 50 percent of women meet the physical activity goal [17].

Physical activity recommendations can be achieved through leisure time physical activity, which can be divided into four exercise categories, including sport, conditioning exercise, household tasks, and other (recreation) [18]. Sport has been defined as “a human activity of achieving a result requiring physical exertion and/or physical skill which, by its nature and organization, is competitive and is generally accepted as being a sport” [19]. Previous research has found that both men and women who participate in sport, compared to the other three exercise categories, have reduced risk of cardiovascular disease, including stroke and heart attack, diabetes, high cholesterol, high blood pressure, some cancers, including skin cancer, chronic obstructive pulmonary disease (COPD), arthritis, kidney disease, and depression [20,21].

Several sociodemographic characteristics affect the frequency and intensity of physical activity. These include age, sex, and ethnicity. In general, studies found that, as age increases, physical activity declines. This is consistent throughout the lifespan—in childhood, adolescence, adulthood, and old age [22,23,24,25,26,27,28]. Women are less likely to engage in physical activity than men [22,29,30,31]. This is especially true for those who belong in minority groups, such as Asian, non-Hispanic blacks, and Hispanics, compared to their non-Hispanic white counterparts [22,29,30,32,33]. However, while both women and men who identify as South Asian have the lowest physical activity levels, in comparison to other ethnic groups, they are also the least sedentary and have low levels of being overweight [29,30].

Other sociodemographic characteristics include marital status, income, employment, and education levels. Some studies found that married individuals spend less time exercising and engaging in moderate-to-vigorous physical activity (MVPA) than those who are not married, where the decrease among married women is more prominent than married men [24,31,34]. However, another study found that spousal pairs had greater involvement in physical activity due to participation in activities together [35]. Children, adolescents, and adults who come from a low-socioeconomic background (<USD20,000) tend to have lower physical activity levels than those who come from a high-income background (>USD75,000). Conversely, those who have a high-economic status are more likely to be physically active [36,37,38,39]. Highly educated individuals—those who have a university degree or a higher vocational schooling—are more likely to have higher levels of physical activity than those who are not as educated. Less educated individuals are likely to rely on employment for activity. Furthermore, they tend to participate in team sports rather than facility-based activities (going to the gym) due to the lack of resources [26,32,40]. Those who are unemployed tend to have lower physical activity levels than those who work full-time [41,42]. However, women with a full-time sedentary job engage in less physical activity than those who do not work [41].

Although a great deal is known about the sociodemographic characteristics of physically active people, particularly when comparing people who are active to those who are not, the sociodemographic characteristics among physically active women, especially of the different subcategories of physical activity (sport, conditioning exercise, recreation, and household tasks) is not known. Because of the added health benefits associated with sport participation, the purpose of this study was to examine the sociodemographic determinants among subcategories of physically active women in the United States by analyzing Behavioral Risk Factor Surveillance System (BRFSS) data.

## 2. Methods

We used data from the 2017 Behavioral Risk Factor Surveillance System (BRFSS) to conduct this secondary data analysis. The BRFSS is the largest cross-section health survey of adults in the United States. It is a collaboration between the Centers for Disease Control and Prevention (CDC) and USA states and territories that began in 1984 [43]. In odd years, participants answer a series of physical activity questions, including type, duration, and frequency. Data from the 2017 BRFSS were used for this study because data from the 2019 survey were not yet available at the time of the data analyses, and data from the 2018 survey did not include the exercise module.

### 2.1. Participants

The BRFSS is a random-digit dial telephone survey of adults over the age of 18 years who are not institutionalized and reside in any U.S. state or territory [43]. In 2017, 450,016 people participated in the BRFSS. To provide a suitable sample size for smaller geographic areas, disproportionate stratified sampling is used [43]. Data are weighted for non-coverage and non-response, making it more generalizable to the population [43]. Detailed information about the BRFSS weighting, sampling, and survey administration can be found at https://www.cdc.gov/brfss.

### 2.2. BRFSS Survey and Variables

The BRFSS survey consists of different components with the core component including the questions that are asked of everyone who participates in the survey. Participants are asked to provide information about their demographics, chronic diseases, preventive health practices, and health/health risk behaviors. In odds years, physical activity questions are included in the core component. Participants are asked about the physical activity that they engaged in over the past month. Specifically, the initial question inquires about any physical activity outside of work that was performed for exercise. [44]. If a participant indicates that they had participated in physical activity other than at work, they are then asked more specific questions about their physical activity. They are asked about the type of physical activity that they spent the most time doing during the past month, as well as how long they did the activity (minutes or hours) and how often they did the activity (days or weeks) [44]. There are 76 different types of physical activity identified by participants that are listed in Table 1. For this study, we examined the physical activity that women reported doing the most and acknowledge that women may have engaged in multiple types of physical activity during the month.

Based on the answers to the physical activity questions (activity type, duration, frequency), the CDC calculates several different variables for physical activity. First, based on the answer to the type of physical activity, the CDC assigns a metabolic equivalency (METs) value to the activity to indicate exercise intensity. Next, physical activity categories (highly active, active, insufficiently active, or inactive) and whether or not the participant met the DHHS recommendations for aerobic exercise are determined based on METs, duration, and frequency. Physical activity levels established by the CDC using BRFSS data are as follows: Highly Active—respondents who participated in 300 min of moderate aerobic activity or 150 min of vigorous aerobic exercise; Active—respondents who participated in 150–300 min of aerobic activity (or the vigorous equivalent); Insufficiently Active—respondents who were physically active for 11–149 min; Inactive—respondents who did less than 11 min of aerobic activity [44]. Participants are also categorized as either meeting the DHHS aerobic exercise recommendations (150+ min of moderate aerobic exercise or vigorous equivalent) or as not meeting aerobic recommendations (less than 150 min of moderate aerobic exercise) [44].

Women who answered “no” or refused to answer the initial physical activity question, who refused to answer the question about the type of physical activity, and men were excluded from our analysis. We used the same methodology for identifying the physical activity categories of sport, conditioning exercise, household tasks, and recreation that we used in two previous studies, as shown in Table 1 [20,21].

### 2.3. Statistical Analyses

We used SAS version 9.3 (SAS Institute Inc., Cary, NC, USA) for the statistical analyses of demographic characteristics by physical activity category. Weighted descriptive statistics were performed to describe the sociodemographic characteristics of the four exercise categories by gender, age, race, education, income, employment, and marital status. For employment, the term ‘out of the labor force’ (OLF) was used to identify participants who were not working but also were not looking for employment. Participants who were OLF were retired, students, or homemakers versus being unemployed. To determine statistically significant differences in sociodemographic characteristics by physical activity category, χ^2^ tests were performed. Chi square tests were also used to determine differences among groups for their physical activity level (highly active, active, insufficiently active, inactive) and whether or not they had met aerobic exercise recommendations. Because the categorical variables had more than two categories, we calculated Cramer’s V statistic for effect size. In instances where significant differences were found between variables in the contingency table, we used the weighted frequency counts to perform multiple comparison post hoc analyses. Additionally, we calculated the mean number of minutes and the mean METs associated with the activity for each of the physical activity categories along with a 95% confidence interval (CI) to compare groups.

## 3. Results

**Figure 1:**
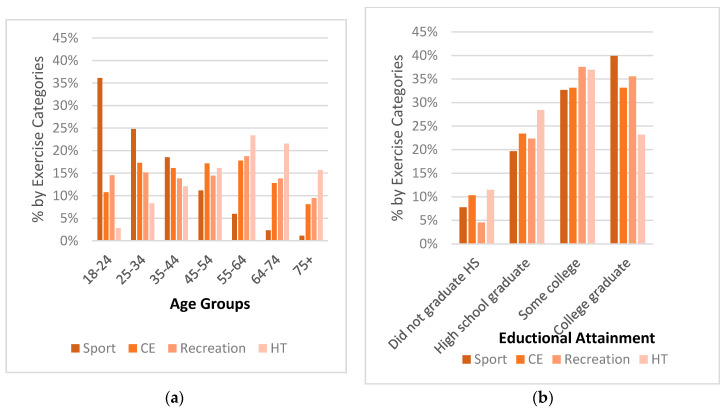

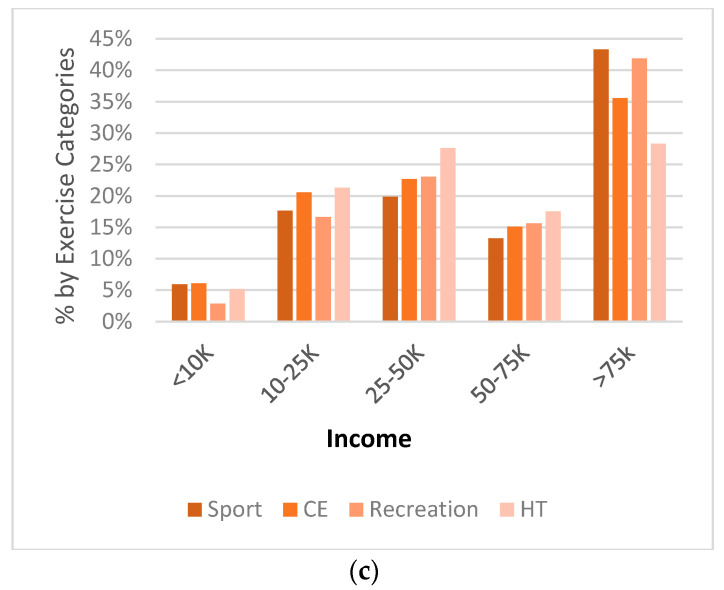
Sociodemographic Percentages by Exercise Categories: (**a**) Age Groups, (**b**) Educational Attainment, (**c**) Income. Conditioning exercise = CE; Household tasks = HT.

**Figure 2:**
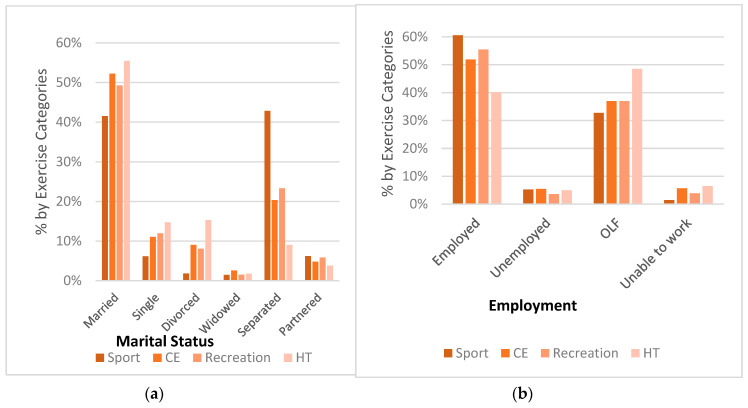
Sociodemographic Percentages for Exercise Categories: (**a**) Marital Status, (**b**) Employment. Conditioning exercise = CE; Household tasks = HT.

A total of 164,948 (70.9%) of the women surveyed reported exercising in the past month with 29.1% of women reporting no exercise. Of the women who exercised, 153,218 indicated that they participated in one of the 76 physical activity categories that were used in this analysis. Of those, 11.7% participated in an activity classified as sport, 78.3% in an activity classified as conditioning exercise, 3.1% in recreation activities, and 6.8% in household tasks. There were significant differences in all sociodemographic variables among the four subcategories of physical activity, as shown in Table 2. Post hoc analyses revealed that there were significant differences between the subcategories of physical activity for every sociodemographic variable level. Women who participated in sport were more likely to be in the younger age groups; however, physical activity declined among all subcategories beyond the age of 64, as shown in Figure 1. Moving from the age bracket of 35–44 to 45–54 and beyond, there is a shift in exercise categories from sport participation to household tasks with household tasks being the prominent form of exercise beyond the age of 55 years. Women who participated in sport were more racially diverse, as shown in Table 2, likely to be employed, and college graduates, compared to the other subcategories, as shown in Figure 1 and Figure 2. Women who were unemployed, lacked a high school diploma, and made less than USD10,000 were the least likely to be represented in any of the four exercise categories. Based on the Cramer’s V statistic, there was a moderate association between physical activity subcategories and age, marital status, physical activity level, and meeting the physical activity guidelines. There was a weak association between physical activity subcategories and educational attainment, race/ethnicity, income, and employment.

Interestingly, women who participated in recreation or household tasks were more likely to be categorized as highly active and to have met the recommended amounts of physical activity, as shown in Table 2. This was accomplished by exercising for a significantly greater number of minutes compared to those who participated in sport or conditioning exercise; however, they exercised at a lower intensity than women who participated in sport, as shown in Table 3.

## 4. Discussion

Because of the health benefits of physical activity and sports participation for women, along with the added health benefits associated with sports participation, the purpose of this study was to understand the sociodemographic characteristics of women who participate in the four subcategories of physical activity, including sport [20]. Interestingly, the sociodemographic characteristics of women who participate in sport mirror the sociodemographic characteristics of women who were found to be physically active in previous research. In general, women who are physically active are younger than women who are inactive and physical activity declines with age. In this study, women who participated in sport were younger than women who participated in the other three exercise categories, and the rates of sport participation declined with age. This may be due to several reasons. Women who participate in sport did so at a higher intensity (METs), and the high intensity of sport may be difficult to maintain as a person ages. Studies among Master’s athletes show that the athletes are able to maintain their exercise intensity until around age 35, then there is a modest decrease in intensity until age 50–60 with progressively steeper declines in intensity after the age of 60 [45]. Reasons for this decrease include lower cardiac output, lower maximal heart rate, decreased oxygen consumption, and decreased stroke volume [45]. The practical implications for public health of these findings include designing sport and physical activity programs with adaptable intensities for women as they age. For example, Ireland’s “Building pathways in Irish Sport” provides recreational pathways to ensure lifelong involvement in sport [46,47].

Women who participated in sport were also less likely to be married and more likely to be single. Some research has found that physical activity is reduced among married women while others have found the contrary [35]. Our research showed that women who were married were more likely to engage in household tasks as a form of physical activity rather than sport. This supports Sobal and Hansen (2010), who found that physical activity among married people was more often task-oriented, such as gardening and yardwork [27]. This may be a result of time demands that come with marriage and, potentially, parenthood, that compete with time for other forms of physical activity [34]. It may be the most efficient use of time to be physically active while completing household tasks. This idea is supported by research which finds that a main reason that adults stop sport participation is due to a lack of time [48]. Married women were more likely to engage in conditioning exercise, which included activities that often occur at a gym. This may be a convenience issue, with it being easier to stop by the gym at a convenient time rather than meeting at a scheduled time for sport practice. Additionally, many gyms offer childcare, making it more convenient than other forms of exercise.

Women who participated in sport and recreation were more likely to be employed and in the higher income brackets, while women who participated in household tasks were more likely to be in the USD25 K–USD50 K income bracket and OLF. This may be due to the cost associated with sport participation and recreational activities. Additionally, research has found that low income neighborhoods have significantly fewer facilities for physical activity as well as fewer free-for-use facilities than high income neighborhoods, limiting the opportunities for women in lower income brackets to participate in sport or recreation [49]. Public health practitioners could use community capacity building strategies, which are defined as the development of knowledge, skills, commitment, structures, systems, and leadership to increase sport participation in low income communities [50]. This strategy was used effectively in Belgium, where there was a significant increase in sport participation in low income communities after a community capacity building program was implemented [51]. Additionally, other European countries have made financial investments to increase participation in mass sporting events, such as running and cycling, to decrease the disparity in participation, based on income, especially among women [46,47,52,53]. Women who were OLF were more likely to engage in household tasks as their form of physical activity, compared to the other exercise categories. Women who are OLF are typically retired and may have more time to complete household tasks, such as gardening. They may also be more inclined to participate in exercise in a lower intensity than sport, for reasons mentioned above.

This study is not without limitations. Because the BRFSS is cross-sectional, causation cannot be determined [54]. Additionally, because the data collected in the BRFSS are self-reported, there is the possibility of self-report bias. Participants may have under or over reported based on their perception of social acceptability [55]. Additionally, the data analyzed in this study only include the most frequent types of physical activity performed. Women may have participated in other types of physical activity in addition to the types analyzed, and we were not able to capture this information, which is a limitation of the study. 

## 5. Conclusions

The sociodemographic characteristics of physical activity and sport participation can be used to create promotional strategies to increase physical activity and sport participation and improve fitness and health among women. Findings from this study have implications for public health practice as we strive to help people make the easy choice a healthy one. Although we only analyzed the physical activity types that women did the most, knowing what they are most likely to do can contribute to this body of literature and have meaning for health promotion. Based on behavior theory, a person is more likely to engage in a behavior that is supported by their environment and that they have the self-efficacy or skills for [56]. A person’s first choice of an activity is often their preferred choice, and women are more likely to continue a physical activity that they choose [56,57]. We can use findings from this study to meet women where they are in terms of promoting physical activity that works best, based on their sociodemographic characteristics. Additionally, public health professionals can develop health promotion programs to increase physical activity and sport participation among women from other sociodemographic groups by ensuring that programs are affordable, accessible, and can accommodate varying intensity levels over their lifespans. This has been done successfully in European countries by providing recreational pathways to ensure lifelong involvement in sport, through investment in mass participation sport, and through community capacity building strategies.

## Figures and Tables

**Table 1:** Exercise Categories for Reported Activities.

Sport	Conditioning Exercise	Recreation	Household Tasks
Badminton	Active Game Device (i.e., Wii)	Backpacking	Carpentry
Basketball	Aerobics class	Boating	Childcare
Bicycling	Bicycle machine	Bowling	Farming/ranching
Boxing	Calisthenics	Canoeing	Gardening
Golf	Dancing	Fishing	Housework (vacuuming)
Handball	Elliptical machine	Frisbee	Mowing lawn
Hockey	Inline skating	Hiking	Painting house
Lacrosse	Jogging	Horseback riding	Raking lawn
Mountain climbing	Karate	Hunting—small and large game	Snow blowing
Racquetball	Pilates	Paddleball	Snow shoveling
Running	Rope skipping	Snorkeling	Yard work
Ruby	Rowing machine	Stream fishing	-
Rock climbing	Scuba diving	Swimming—not laps	-
Soccer	Skateboarding	Table tennis	-
Softball/baseball	Ice-skating	Waterskiing	-
Squash	Snow skiing	-	-
Tennis	Snowshoeing	-	-
Touch football	Stairmaster	-	-
Volleyball	Surfing	-	-
Wrestling	Swimming—laps	-	-
-	Tai chi	-	-
-	Walking	-	-
-	Weight-lifting	-	-
-	Upper body cycling	-	-

**Table 2:** Demographic Characteristics by Exercise Category—Weighted Percentages.

Variables	Total	Sport	CE	Recreation	HT	X^2^, *p*-Value, and Cramer’s V
	%	Weighted %	Weighted %	Weighted %	Weighted %	
		11.70%	78.30%	3.10%	6.80%	
**Marital status**					**1467**, ***p* < 0.01**, **0.124**	
Married	51.10%	41.50%	52.24%	49.26%	55.43%	
Single	10.76%	6.15%	11.06%	11.98%	14.71%	
Divorced	8.56%	1.82%	9.01%	8.09%	15.28%	
Widowed	2.35%	1.46%	2.57%	1.50%	1.75%	
Separated	22.31%	42.85%	20.35%	23.32%	9.03%	
Partnered	4.91%	6.22%	4.77%	5.84%	3.79%	
**Educational Attainment**					**715**, ***p* < 0.01**, **0.059**	
Did not graduate HS	9.92%	7.76%	10.33%	4.53%	11.43%	
High school graduate	23.27%	19.67%	23.40%	22.35%	28.41%	
Some college	33.48%	32.69%	33.14%	37.56%	36.96%	
College graduate	33.32%	39.89%	33.14%	35.56%	23.20%	
**Age**					**3061**, ***p* < 0.01**, **0.186**	
18–24	13.31%	36.13%	10.73%	14.52%	2.83%	
25–34	17.51%	24.80%	17.30%	15.16%	8.34%	
35–44	16.07%	18.52%	16.14%	13.84%	12.07%	
45–54	16.29%	11.13%	17.15%	14.43%	16.13%	
55–64	16.78%	5.96%	17.76%	18.76%	23.39%	
64–74	12.19%	2.33%	12.80%	13.80%	21.55%	
75+	7.85%	1.12%	8.12%	9.49%	15.69%	
**Race/ethnicity**					**377**, ***p* < 0.01**, **0.068**	
White	65.82%	59.86%	65.09%	77.16%	79.41%	
Black	10.84%	9.23%	11.78%	4.38%	5.80%	
Hispanic	14.69%	19.79%	14.61%	10.34%	8.71%	
AI/NA	0.92%	0.76%	0.92%	1.09%	1.10%	
Asian	5.67%	8.15%	5.58%	5.51%	2.52%	
NH/PI	0.15%	0.20%	0.15%	0.11%	0.08%	
Other	0.42%	0.33%	0.41%	0.15%	0.84%	
Multiple	1.48%	1.68%	1.45%	1.26%	1.55%	
**Income**					**150**, ***p* < 0.01**, **0.045**	
<10 K	5.90%	5.91%	6.08%	2.83%	5.18%	
10–25 K	20.17%	17.66%	20.58%	16.64%	21.31%	
25–50 K	22.69%	19.86%	22.67%	23.03%	27.64%	
50–75 K	15.07%	13.26%	15.11%	15.62%	17.56%	
>75 k	36.18%	43.31%	35.55%	41.88%	28.31%	
**Employment**					**300**, ***p* < 0.01**, **0.059**	
Employed	52.20%	60.55%	51.89%	55.52%	40.10%	
Unemployed	5.35%	5.24%	5.48%	3.60%	4.93%	
OLF	37.28%	32.75%	36.99%	36.99%	48.52%	
Unable to work	5.16%	1.45%	5.65%	3.90%	6.46%	
**Physical Activity Level**					**913**, ***p* < 0.01**, **0.116**	
Highly active	40.87%	45.39%	36.72%	62.80%	70.33%	
Active	27.29%	29.55%	28.12%	21.23%	16.44%	
Insufficiently active	29.75%	24.17%	32.76%	14.31%	12.11%	
Inactive	2.10%	0.89%	2.39%	1.66%	1.12%	
**Aerobic exercise recommendations**					**483**, ***p* < 0.01**, **0.144**	
Met aerobic recommendations	68.42%	75.09%	65.14%	84.12%	86.94%	
Did not meet aerobic recommendations	31.58%	24.91%	34.86%	15.88%	13.06%	

Conditioning exercise = CE; Household tasks = HT; OLF = out of labor force; AI/NA = American Indian/Native Alaskan; NH/PI = Native Hawaiian/Pacific Islander.

**Table 3:** Exercise Minutes and Metabolic Equivalence (METs) by Exercise Type.

Variable	Sport Mean (95% CI)	CE Mean (95% CI)	Recreation Mean (95% CI)	HT Mean (95% CI)
Minutes of Exercise	207.64 (198.27–217.00)	192.86 (189.20–196.52)	256.44 (242.15–270.73)	450.34 (425.64–475.05)
METs	6.23 (6.20–6.25)	3.71 (3.68–3.73)	5.34 (5.21–5.47)	4.76 (4.74–4.78)

Conditioning exercise = CE; Household tasks = HT; CI = Confidence Interval.
